# Surgical Treatment of Acute Type A Aortic Dissection with 18-Litre Bleeding

**DOI:** 10.1055/s-0041-1722897

**Published:** 2021-10-04

**Authors:** Sergey Y. Boldyrev, Kirill O. Barbukhatty, Vladimir A. Porhanov

**Affiliations:** 1 Department of Cardiac Surgery no. 2, S.V. Ochapowski Regional Hospital no. 1, Krasnodar, Russia; 2 Kuban State Medical University, Krasnodar, Russia

**Keywords:** aorta, dissection, acute, bleeding

## Abstract

Surgical treatment of Type-A acute aortic dissection is associated with high mortality and morbidity. One of the reasons is perioperative bleeding, which may lead to worse outcomes. We present a case of successful treatment of a patient with 18-litre perioperative blood loss in DeBakey Type-I acute aortic dissection with drug-induced hypocoagulation and malperfusion of a lower extremity.

## Introduction

In spite of improvement of surgical treatment, Type-A acute aortic dissection (AAAD) is so far associated with relatively high mortality. Low incidence and diversity of clinical manifestations of AAAD sometimes lead to wrong diagnosis. Coagulopathy developing in the settings of the main disease 1 is significantly compromised by unreasonably administered antiaggregants and anticoagulants that leads inevitably to the increase of risk of perioperative bleeding as one of the most dangerous life-threatening complications. 2 3 Surgical aggression, malperfusion syndrome, duration of procedure, blood loss, and many other factors inevitably decrease the patients' survival chances. In this article, we would like to share our experience of successful treatment of a patient with 18-L perioperative blood loss in DeBakey Type-I acute aortic dissection with drug-induced hypocoagulation and malperfusion of a lower extremity.

## Case Presentation

A 59-year-old man was urgently hospitalized in September 2017 with complaints of chest pain, a pain in the left lower extremity and numbness in the left foot. According to the patient, he had been injected with nonsteroidal anti-inflammatory drugs in unknown doses by an emergency response physician (it was not documented in referral medical documents). Computed tomography angiogram of the chest was performed and demonstrated dilatation of the ascending aorta with DeBakey Type-I dissection and radiological signs of asymptomatic dissection of the brachiocephalic trunk, left common carotid, right coronary and left renal arteries, and common iliac and common femoral arteries on the left side. Echocardiography showed a DeBakey Type-I aortic dissection and dilatation of ascending aorta to 49-mm, tricuspid aortic valve (AV) with regurgitation ++ + . Left side of the heart was enlarged with preserved ejection fraction (EF). Triplex sonography (TS) of the lower extremities' arteries showed absence of blood flow in the major arteries distal to the left common femoral artery. The electrocardiogram (ECG) was without any ischemic sings and coronary angiography demonstrated intact coronary arteries. Laboratory results showed decreased platelet aggregation with adenosine 5′-diphosphate to 31%; creatine kinase was increased to 314 U/L. The patient underwent a two-stage surgical procedure.

**Fig. 1:**
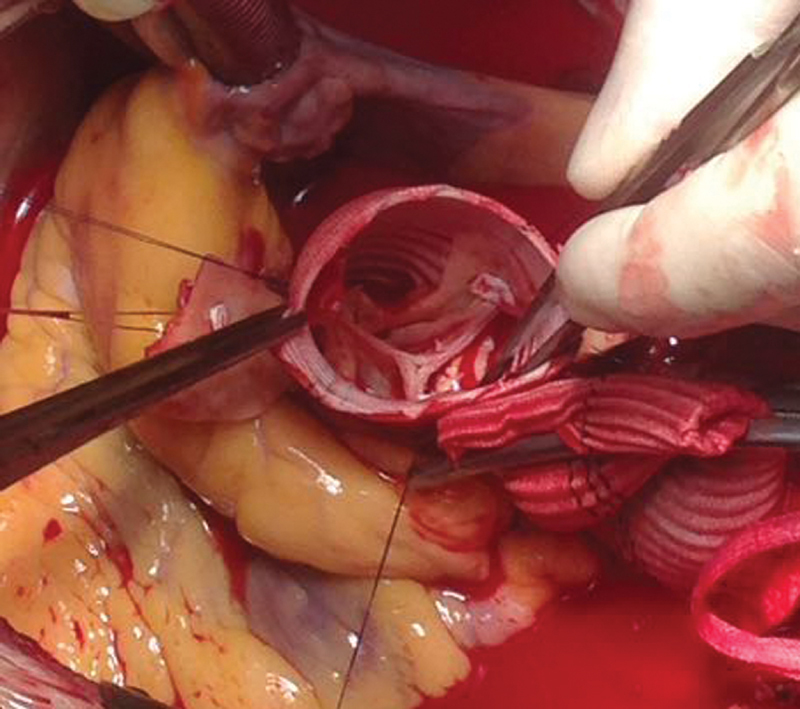
The reimplanted aortic valve.

**Fig. 2:**
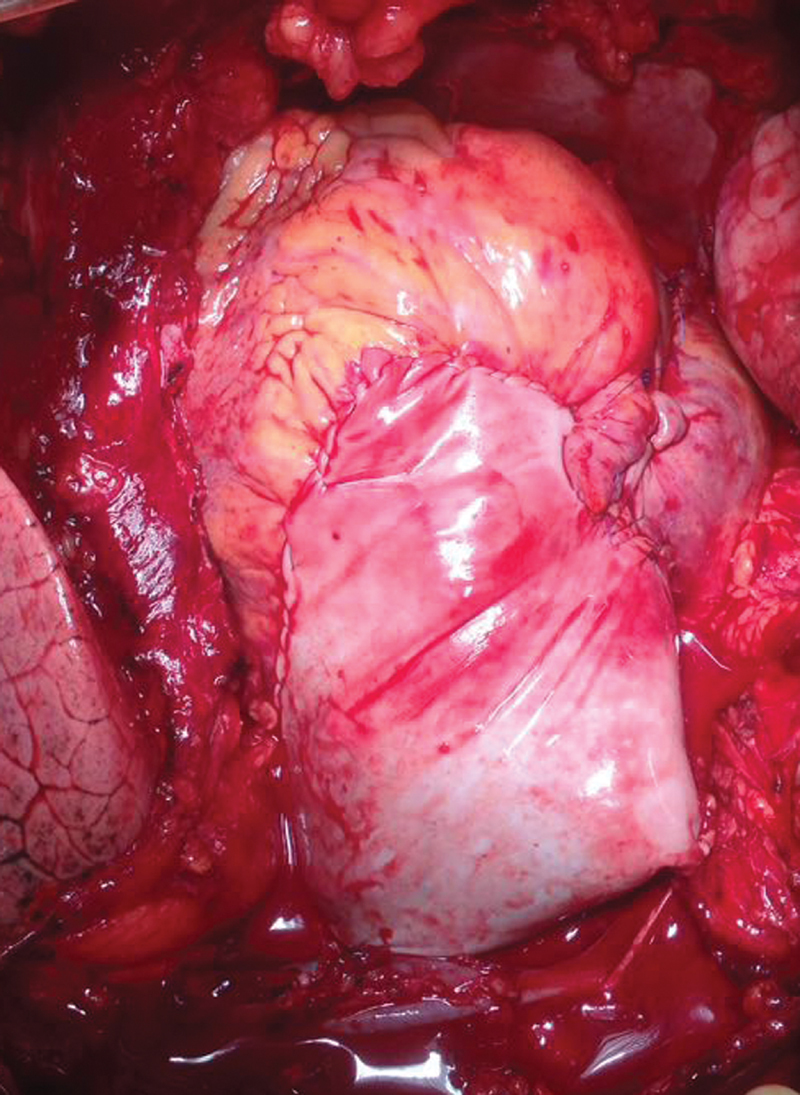
The modified Cabrol shunt between the perigraft space to right atrial appendage using a bovine pericardial patch.

As a first stage, a cross right-to-left femoral-femoral bypass with an Intergard heparin-knitted 10 mm graft, was performed. After the blood flow restarted, pulsation in the major arteries of the left lower extremity was detected on palpation along the entire length. The TS of the lower extremity arteries was performed intraoperatively; the blood flow in the arteries of the right and left lower extremities was satisfactory. By the end of the first stage, acid-base imbalance in the blood was revealed. The diagnostics of ischemia-reperfusion injury markers was performed, the creatine phosphokinase level reached 1,544.70 U/L; the MB fraction was normal. Taking into account the admissible values of laboratory results and the absence of growth of biochemical markers of tissue injury, repair of the ascending aorta and aortic arch using the INTERGARD vascular graft was performed as the second stage. Distal “hemiarch” anastomosis and reimplantation of the AV into the neoaorta proximally ( Fig. 1 ) were performed. 4 The bypass time was 320 minutes, cross-clamp time was 167 minutes, and duration of circulatory arrest was 29 minutes. After administration of protamine sulfate, persistent oozing and bleeding was observed at the sites of the vascular implant punctures and along the suture lines. After prolonged and unsuccessful hemostasis, the modified Cabrol shunt was performed between the perigraft space to right atrial appendage using a bovine pericardial patch ( Fig. 2 ). The transverse sinus was blocked with local hemostatic materials.

The total operative time was 8 hours and intraoperative blood loss was 9 L. In the end of procedure, pericardium, anterior mediastinum, and both pleural cavities were drained. In the early postoperative period the bleeding was continuing, its correction required massive transfusion therapy with 6,700 mL of packed red blood cells, 6,600 mL of fresh frozen plasma, and 600 mL of platelets. Using a cell saver, 5,200 mL of autologous blood was returned. The total volume of blood loss in first 6 hours after surgery was 9 L. After intensive conservative therapy, the rate of bleeding decreased from 1,000 to 200 mL per hour. The drainage tubes were removed on day 6. The late postoperative period was complicated by prolonged (20 days) intubation and renal failure. On day 44 after surgery, the patient was discharged in satisfactory condition. The patient was followed-up 1 year after surgery: he was active and did not have any complaints, but he was limping on the left leg. Good AV function and preserved EF of left ventricle were observed.

## Discussion

Early initiation of antiplatelet therapy is definitely recommended to reduce mortality in patients with acute coronary syndrome (ACS). Unfortunately for aortic surgeons, ACS cannot always be confirmed by specific ECG changes, which increases the level of cardiac biomarkers, so one-third of AAAD cases get dual antiplatelet therapy 5 6 and a surgeon faces a situation of impossibility of complete hemostasis.

The current guidelines do not contain sufficient information regarding management of the patients with AAAD in the settings of antiplatelet agent exposure. The drug-induced platelet dysfunction together with hemodilution, total heparinization, hypothermia, metabolic acidosis, allogeneic blood product transfusion, malperfusion syndrome, etc., significantly compromises consumption coagulopathy. 1 2 3 6 7

Such difficult cases, including those presented here, require high concentration of medical team to develop an individual multicomponent treatment approach, especially in blood loss control. Taking into account the fact the manifestation of consumption coagulopathy occurs in the first hours of acute dissection, it requires an increased volume of blood components in the perioperative period. So, according to data of Danish authors, 5 in drug-induced hypocoagulation among patients with acute aortic syndrome, the requirement of blood components markedly increases. We compared our conventional data on transfusion supply of patients who underwent surgery for AAAD with data presented by Chemtob et al. 5 The volume of blood components that we used was several times less. Even though such surgical techniques as various modification of Cabrol's shunt 8 have significantly improved the control of bleeding they, in our opinion, do not always guarantee sufficient hemostasis, especially in patients with coagulopathy.

In spite of apparentness of this unsolved problem, centers, and clinics performing urgent surgeries of aorta will still have to work in complicated conditions of inadequate supply of materials for coagulopathy control. Although coagulopathy and high rate of intraoperative mortality are inevitably associated with acute aortic dissection, the surgery is the only survival chance for a patient.
